# Taxonomic revision of the Malagasy *Nesomyrmex madecassus* species-group using a quantitative morphometric approach

**DOI:** 10.3897/zookeys.603.8271

**Published:** 2016-07-06

**Authors:** Sándor Csősz, Brian L. Fisher

**Affiliations:** 1 Entomology, California Academy of Sciences, 55 Music Concourse Drive, San Francisco, CA 94118, U.S.A.

**Keywords:** Madagascar, taxonomy, morphometry, species delimitation, exploratory analyses, gap statistic, biogeography

## Abstract

Here we reveal the diversity of the next fragment of the Malagasy elements of the ant genus *Nesomyrmex* using a combination of advanced exploratory analyses on quantitative morphological data. The diversity of the *Nesomyrmex madecassus* species-group was assessed via hypothesis-free *nest centroid clustering* combined with *recursive partitioning* to estimate the number of clusters and determine the most probable boundaries between them. This combination of methods provides a highly automated species delineation protocol based on continuous morphometric data, and thereby it obviates the need of subjective interpretation of morphological patterns. Delimitations of clusters recognized by these exploratory analyses were tested via confirmatory Linear Discriminant Analysis (LDA). Our results suggest the existence of four morphologically distinct species, *Nesomyrmex flavus* **sp. n.**, *Nesomyrmex gibber*, *Nesomyrmex madecassus* and *Nesomyrmex nitidus* **sp. n.**; all are described here and an identification key for their worker castes using morphometric data is given. Two members of the newly outlined *madecasus* species-group, *Nesomyrmex flavus* **sp. n.** and *Nesomyrmex nitidus* **sp. n.**, represent true cryptic species. Geographic maps depicting species distributions and elevational information for the sites where populations of particular species were collected are also provided.

## Introduction

The ant fauna of the Malagasy zoogeographical region, i.e. Madagascar and its surrounding islands (Bolton 1994), has recently been the subject of intensive systematic research (Fisher 2009, Blaimer and Fisher 2013, Yoshimura and Fisher 2012, Hita-Garcia and Fisher 2014). Thanks to these efforts to explore Malagasy biodiversity, our knowledge of the island’s myrmecofauna has increased considerably. These latest findings support earlier assumptions about the high species diversity of the region. The goal of the current paper is to contribute to this endeavor and clarify the taxonomy of another segment of the Malagasy *Nesomyrmex* fauna, the *Nesomyrmex madecassus* species-group.

The four species in this group are known to nest in small diameter (pencil size) dead twigs above ground. They can be found foraging on tree trunks and occasionally in the leaf litter at higher elevations. There is also the occasional record of nests in rotten logs at higher elevations. But in general, to collect these species, the best approach is to break open small dead twigs. We know little of their biology but field observations suggest they are generalist scavengers. Morphological diversity is assessed via a taxonomic protocol NC-PART clustering introduced by Csősz and Fisher (2016a, 2016b) based on multivariate analyses of quantitative morphological data. This method incorporates elements of NC-clustering (Seifert et al. 2014) and the partitioning algorithms known as ‘part’ (Nilsen et al. 2013). Benefits of the combined application of Nest Centroid clustering (NC clustering) and Partitioning Algorithm based on Recursive Thresholding (PART) was described in detail in Csősz and Fisher (2016a, 2016b) and its efficiency in species delimitation has proven in two *Nesomyrmex* species-groups and in a fragment of the Malagasy *Camponotus* fauna (Rakotonirina et al. 2016). The NC clustering searches for discontinuity in morphometric data by sorting all similar cases into clusters in a two-step procedure. This technique has proved efficient at pattern recognition within large and complex datasets, but the number of clusters is still subjectively defined based on the obtained dendrogram. The partitioning method PART allows for estimation on the number of clusters via recursive application of the Gap statistic (Tibshirani et al. 2001) algorithm and automated assignment of each sample in either clusters.

Multivariate evaluation of morphological data has revealed that the *Nesomyrmex madecassus* species-group incorporates four well-outlined clusters in the Malagasy zoogeographical region, all representing species. Two of them, *Nesomyrmex gibber* (Donisthorpe, 1946) and *Nesomyrmex madecassus* (Forel, 1892) are already described taxa, but two new species, *Nesomyrmex flavus* sp. n. and *Nesomyrmex nitidus* sp. n., are being described here based on worker caste. The latter two species represent true cryptic species (Seifert 2009) which can be convincingly separated by using a combination of morphometric data. We provide a combined key that uses a traditional, character-based key, and a separation of the two cryptic taxa, *Nesomyrmex flavus* sp. n. and *Nesomyrmex nitidus* sp. n. is supported by a character combination. Morphological patterns are linked to geographic map elevations of the sites where populations were collected and are also provided as predictor variables.

## Material and methods

The group was defined earlier by Csősz and Fisher (2015) as one of the four remarkable lineages occurring in the region, and defined as follows: “Pronotal spines absent. Anterodorsal spines on petiolar node absent. Propodeal spines short, lamelliform to absent. Vertex ground sculpture smooth. Vertex main sculpture not defined. Metanotal depression present. Median clypeal notch present or absent. Median clypeal notch shape/depth 0–15 µm. Antennomere count: 12. Absolute cephalic size (CS): 571 µm [405, 785]. Cephalic length vs. maximum width of head capsule (CL/CWb): 1.231 [1.092, 1.567]. Postocular distance vs. cephalic length (PoOc/CL): 0.479 [0.407, 0.544]. Scape length vs. absolute cephalic size (SL/CS): 0.718 [0.492, 0.831]. Eye length vs. absolute cephalic size (EL/CS): 0.249 [0.1934, 0.279]. Petiole width vs. absolute cephalic size (PEW/CS): 0.217 [0.181, 0.256]. Postpetiole width vs. absolute cephalic size (PPW/CS): 0.331 [0.243, 0.398]. Petiolar node height vs. absolute cephalic size (PEW/CS): 0.122 [0.072, 0.158].

In the present study, 18 continuous morphometric traits were recorded in 231 worker individuals belonging to 172 nest samples collected in the Malagasy region.

The material is deposited in the following institutions, abbreviations after Evenhuis (2013): CASC (California Academy of Sciences, San Francisco, California, U.S.A.), MCZ (Museum of Comparative Zoology, Cambridge, Massachusetts, U.S.A.), MHNG (Muséum d’Histoire Naturelle, Geneva, Switzerland) and Phil S. Ward’s collection (University of California Davis Davis, California, U.S.A.).

All images and specimens used in this study are available online on AntWeb (http://www.antweb.org). Images are linked to their specimens via the unique specimen code affixed to each pin (CASENT0101667). Online specimen identifiers follow this format: http://www.antweb.org/specimen/CASENT0101667.

Digital color montage images were created using a JVC KY-F75 digital camera and Syncroscopy Auto-Montage software (version 5.0), or a Leica DFC 425 camera in combination with the Leica Application Suite software (version 3.8). Distribution maps were generated in R (R Core Team 2015) via ‘phylo.to.map’ function using package phytools (Revell 2012).

The measurements were taken with a Leica MZ 12.5 stereomicroscope equipped with an ocular micrometer at a magnification of 100×. Measurements and indices are presented as arithmetic means with minimum and maximum values in parentheses. Body size dimensions are expressed in µm. Due to the abundance of worker individuals available relative to queen and male specimens, the present revision is based on worker caste only. Worker-based revision is further facilitated by the fact that the name-bearing type specimens of the vast majority of existing ant taxa belong to the worker caste. All measurements were made by the first author. For the definition of morphometric characters, earlier protocols (Csősz et al. 2015, Csősz and Fisher 2015, 2016a, 2016b) were considered. Explanations and abbreviations for measured characters are as follows:

CL
 Maximum cephalic length in median line. The head must be carefully tilted to the position providing the true maximum . Excavations of hind vertex and/or clypeus reduce CL.

CW
 Maximum width of the head . Includes compound eyes.

CWb
 Maximum width of head capsule without the compound eyes . Measured just posterior of the eyes.

CS
 Absolute cephalic size . The arithmetic mean of CL and CWb.

EL
 Maximum diameter of the compound eye .

FRS
 Frontal carina distance . Distance of the frontal carinae immediately caudal of the posterior intersection points between frontal carinae and the torular lamellae. If these dorsal lamellae do not laterally surpass the frontal carinae, the deepest point of scape corner pits may be taken as the reference line. These pits take up the inner corner of the scape base when the scape is directed caudally and produces a dark triangular shadow in the lateral frontal lobes immediately posterior to the dorsal lamellae of the scape joint capsule.

ML (Weber length)
 Mesosoma length from caudalmost point of propodeal lobe to transition point between anterior pronotal slope and anterior pronotal shield . Preferentially measured in lateral view; if the transition point is not well defined, use dorsal view and take the center of the dark-shaded borderline between pronotal slope and pronotal shield as anterior reference point. In gynes: length from caudalmost point of propodeal lobe to the most distant point of steep anterior pronotal face.

MW
 Mesosoma width . In workers MW is defined as the longest width of the pronotum in dorsal view excluding the pronotal spines.

MPST
 Maximum distance from the center of the propodeal stigma to the anteroventral corner of the ventrolateral margin of the metapleuron .

NOH
 maximum height of the petiolar node . Measured in lateral view from the uppermost point of the petiolar node perpendicular to a reference line extending from the petiolar spiracle to the imaginary midpoint of the transition between dorso-caudal slope and dorsal profile of caudal cylinder of the petiole.

NOL
 Length of the petiolar node . Measured in lateral view from the center of petiolar spiracle to dorso-caudal corner of caudal cylinder. Do not erroneously take as the reference point the dorso-caudal corner of the helcium, which is sometimes visible.

PEH
 maximum petiole height . The chord of the ventral petiolar profile at node level is the reference line perpendicular to the line describing the maximum height of petiole.

PEL
 Diagonal petiolar length in lateral view; measured from anterior corner of subpetiolar process to dorso-caudal corner of caudal cylinder .

PEW
 Maximum width of petiole in dorsal view . Nodal spines are not considered.

PoOC
 Postocular distance . Use a cross-scaled ocular micrometer and adjust the head to the measuring position of CL. Caudal measuring point: median occipital margin; frontal measuring point: median head at the level of the posterior eye margin.

PPH
 Maximum height of the postpetiole in lateral view . Measured perpendicularly to a line defined by the linear section of the segment border between dorsal and ventral petiolar sclerite.

PPL
 Postpetiole length . The longest anatomical line that is perpendicular to the posterior margin of the postpetiole and is between the posterior postpetiolar margin and the anterior postpetiolar margin.

PPW
 Postpetiole width . Maximum width of postpetiole in dorsal view.

SL
 Scape length . Maximum straight line scape length excluding the articular condyle.

In verbal descriptions of taxa based on external morphological traits, recent taxonomic papers (Csősz and Fisher 2015, 2016) were considered. Definitions of surface sculpturing are linked to Harris (1979). Body size is given in µm, means of morphometric ratios as well as minimum and maximum values are given in parentheses with up to three digits. Inclinations of pilosity given in degrees. Definitions of species-groups as well as descriptions of species are surveyed in alphabetic order.

**Statistical framework—hypothesis formation and testing.** The present statistic framework follows the procedure applied in Csősz and Fisher (2016a, 2016b). Advantages and limitations of the present procedure are discussed there.

*Generating prior species hypotheses via the combined application of NC clustering and PART.* This method searches for discontinuities in continuous morphometric data and sorts all similar cases into the same cluster in a two-step procedure. The first step reduces dimensionality in data with cumulative linear discriminant analysis (LDA) using nest samples (i.e. individuals collected from the same nest are assumed genetically closely related, often sisters) as groups (Seifert et al. 2014). The second step calculates pairwise distances between samples using LD scores as input and the distance matrix is displayed in a dendrogram. The NC-clustering was done via packages *cluster* (Maechler et al. 2014) and *MASS* (Venables and Ripley 2002).

*The ideal number of clusters* was determined by Partitioning Algorithm based on Recursive Thresholding via the package clusterGenomics (Nilsen and Lingjaerde 2013) using the function ‘part’, which also assigns observations (i.e. specimens, or samples) into partitions. The method estimates the number of clusters in a data based on recursive application of the Gap statistic (Tibshirani et al. 2001) and is able to discover both top-level clusters as well as sub-clusters nested within the main clusters. If more than one cluster is returned by the Gap statistic, it is re-optimized on each subset of cases corresponding to a cluster until a stopping threshold is reached or the subset under evaluation has less than 2*minSize cases (Nilsen et al. 2013). Two clustering methods, “hclust” and “kmeans” are used to determine the optimal number of clusters with 1000 bootstrap iterations. The results of PART are mapped on the dendrogram by colored bars via function ‘mark.dendrogram’ found in (Beleites and Sergo 2015). The script written in R and can be found in Supporting Information. The script is published by Csősz and Fisher (2016a, 2016b) and is freely accessible.

*Arriving at final species hypothesis using confirmatory Linear Discriminant Analysis (LDA) and LDA ratio extractor.* To provide increased reliability of species delimitation, hypotheses on clusters and classification of cases via exploratory processes were confirmed by LDA
Leave-one-out cross-validation (LOOCV). Classification hypotheses were imposed for all samples congruently classified by partitioning methods while wild-card settings (i.e. no prior hypothesis imposed on its classification) were given to samples that were incongruently classified by the two methods or proved to be outliers.

*Interpreting discriminant functions as identification tools.* In this paper discriminant function analysis is used to determine which variables discriminate between two or more cryptic species. The discriminant functions (D2 and D4) provided in the key and differential diagnoses offer moderately time consuming but accurate opportunities to identify every single individual. The linear equation of the discriminant functions are as follows: *D*_m_ = a_1_*x_1_ + a_m_*x_m_ + c, where *c* is a constant, *a*_1_ through *a*_m_ are the characters in micrometer and *x*_1_ and *x*_m_ are coefficients. The equation must be calculated with the trait names (e.g. SL) substituted with the length of the corresponding traits in micrometer (e.g. 625). The dimensionless number (*D*_m_) returned by the equation must fit either of the species’ scores showing the identity of that particular individual.

## Results

**Figure 1.**
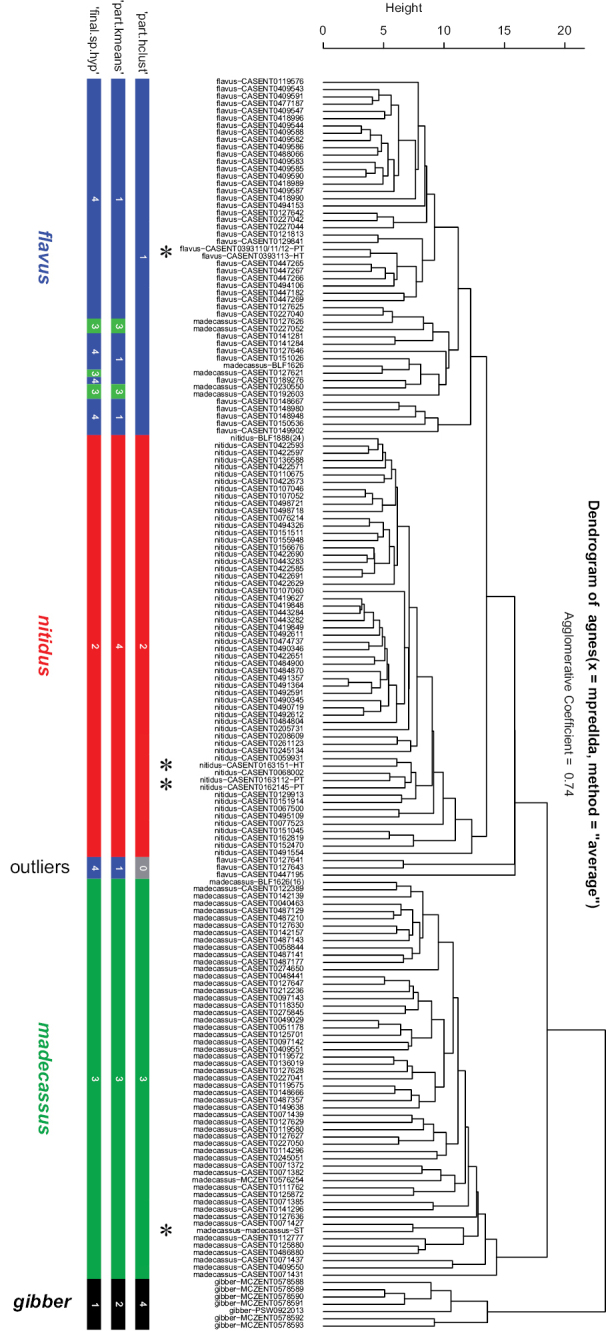
Dendrogram solution for *Nesomyrmex madecassus* species-group. Sample information in the dendrogram follows this format: final species hypothesis followed by CASENT number separated by a hyphen. Three columns of rectangles represent prior species hypothesis resulted by method PART using two cluster methods ‘hclust’ and ‘kmeans’ (for further information see text). Final species hypothesis bar shows classification of samples after confirmation by cross-validated LDA. Different colors distinguish species. *Nesomyrmex flavus* sp. n.: blue, *Nesomyrmex gibber*: black, *Nesomyrmex madecassus*: green, *Nesomyrmex nitidus* sp. n.: red. Outliers returned by ‘part-hclust’ appear in grey. Types are marked by asterisk.

**Figure 2.**
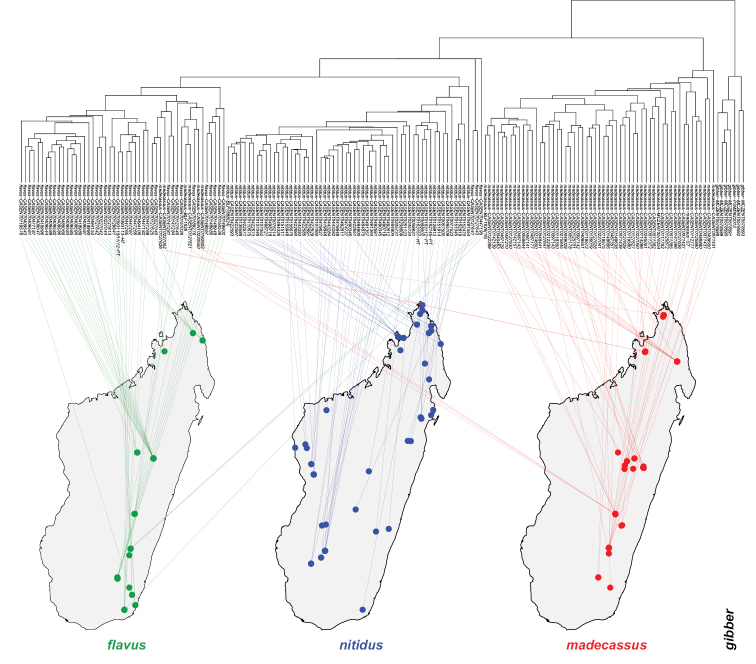
Dendrogram to geographic map. Dendrogram solution is linked on the map of Madagascar. Color codes for species are as follows: *Nesomyrmex flavus* sp. n.: green, *Nesomyrmex gibber*: black, *Nesomyrmex madecassus*: red, *Nesomyrmex nitidus* sp. n.: blue. Samples of *Nesomyrmex gibber* found in Mauritius, East to Madagascar (not shown).

**Figure 3.**
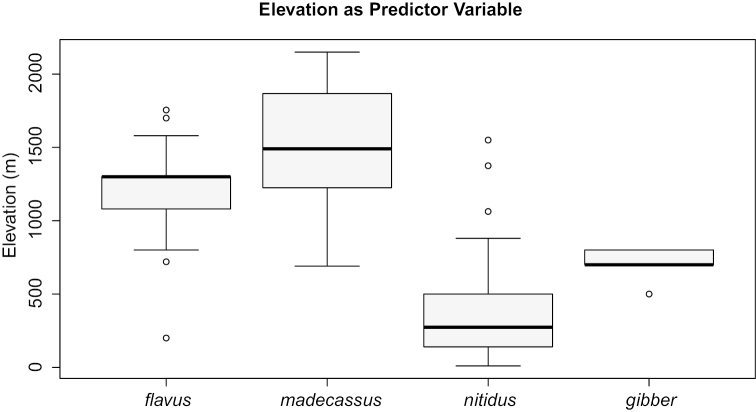
Boxplot for elevational distribution of *Nesomyrmex madecassus* group species. Black line: median, grey box: upper and lower quartiles, whisker: minimum, maximum values, open circles: outliers.

Altogether, four remarkable clusters were recognized by both clustering algorithms “hclust” and ‘kmeans’ using function ‘part’. The pattern returned by these partitioning algorithms can be fitted on the hierarchical structure seen on the dendrogram generated by NC clustering (Fig. 1). The grouping hypotheses generated by the combination of hypothesis-free exploratory analyses was validated by Linear Discriminant Analysis with leave-one-out cross-validation (LOOCV-LDA). The overall classification success is 98% (Table 1), hence the four clusters solution is accepted as the final species hypothesis. The four species described here are as follows in alphabetic order: *Nesomyrmex flavus* sp. n., *Nesomyrmex gibber* (Donisthorpe, 1946), *Nesomyrmex madecassus* (Forel, 1892) and *Nesomyrmex nitidus* sp. n.. Two of the four morphologically diagnosable OTUs, *gibber* and *madecassus*, differ in many qualitative characters (e.g. shape of propodeal spines, petiolar node, surface sculpturing etc.), but the two others, *flavus* and *nitidus*, represent true cryptic species in the sense of Seifert (2009). Morphometric data for species calculated on individuals are given in Table 2. Three of four species, *Nesomyrmex flavus* sp. n., *Nesomyrmex madecassus* (Forel, 1892) and *Nesomyrmex nitidus* sp. n. occur in Madagascar exhibiting different but overlapping geographic distribution (Fig. 2) and elevational ranges (Fig. 3). *Nesomyrmex gibber* is known to occur only in Mauritius.

**Table 1.** Classification matrix obtained by Leave One Out Cross Validation LDA. The last column (percent.correct) shows the classification success in percentage.

	flavus	gibber	madecassus	nitidus	percent.correct
flavus	59	0	2	0	96.7
gibber	0	7	0	0	100
madecassus	2	0	82	0	96.7
nitidus	0	0	0	79	100

**Table 2.** Mean of morphometric ratios calculated species-wise on individual level. Morphometric traits are divided by absolute cephalic size (CS), ±SD are provided in the upper row, minimum and maximum values are given in parentheses in the lower row.

	flavus (n = 61)	gibber (n = 7)	madecassus (n = 84)	nitidus (n = 79)
CS	602 ±35	724 ±33	692 ±37	496 ±26
	[533, 699]	[655, 752]	[616, 763]	[460, 574]
CL/CW	1.21 ±0.04	1.11 ±0.02	1.15 ±0.02	1.19 ±0.03
	[1.15, 1.31]	[1.09, 1.13]	[1.10, 1.20]	[1.12, 1.31]
CL/CWb	1.26 ±0.04	1.17 ±0.02	1.18 ±0.02	1.23 ±0.03
	[1.19, 1.36]	[1.14, 1.18]	[1.13, 1.22]	[1.16, 1.35]
POoC/CL	0.48 ±0.01	0.41 ±0.01	0.46 ±0.01	0.48 ±0.01
	[0.46, 0.50]	[0.39, 0.42]	[0.43, 0.48]	[0.46, 0.50]
FRS/CS	0.30 ±0.01	0.33 ±0.01	0.31 ±0.01	0.31 ±0.01
	[0.28, 0.32]	[0.32, 0.34]	[0.29, 0.33]	[0.29, 0.33]
SL/CS	0.80 ±0.02	0.80 ±0.01	0.78 ±0.02	0.74 ±0.02
	[0.76, 0.83]	[0.78, 0.82]	[0.72, 0.82]	[0.69, 0.78]
EL/CS	0.25 ±0.01	0.25 ±0.01	0.26 ±0.01	0.26 ±0.01
	[0.23, 0.27]	[0.24, 0.26]	[0.24, 0.28]	[0.23, 0.27]
MW/CS	0.60 ±0.02	0.64 ±0.01	0.62 ±0.02	0.60 ±0.01
	[0.57, 0.66]	[0.63, 0.65]	[0.56, 0.66]	[0.57, 0.63]
PEW/CS	0.22 ±0.01	0.21 ±0.01	0.22 ±0.01	0.22 ±0.01
	[0.21, 0.24]	[0.20, 0.23]	[0.19, 0.24]	[0.19, 0.24]
PPW/CS	0.35 ±0.01	0.30 ±0.02	0.35 ±0.02	0.33 ±0.02
	[0.33, 0.40]	[0.27, 0.32]	[0.29, 0.39]	[0.30, 0.36]
ML/CS	1.38 ±0.04	1.41 ±0.01	1.35 ±0.04	1.31 ±0.03
	[1.29, 1.50]	[1.39, 1.42]	[1.26, 1.45]	[1.25, 1.41]
PEL/CS	0.53 ±0.02	0.50 ±0.03	0.50 ±0.02	0.51 ±0.02
	[0.48, 0.57]	[0.46, 0.53]	[0.44, 0.55]	[0.47, 0.58]
NOL/CS	0.35 ±0.02	0.33 ±0.01	0.33 ±0.02	0.34 ±0.02
	[0.30, 0.39]	[0.32, 0.34]	[0.28, 0.38]	[0.31, 0.39]
MPST/CS	0.44 ±0.01	0.46 ±0.01	0.44 ±0.02	0.43 ±0.02
	[0.41, 0.47]	[0.45, 0.47]	[0.41, 0.49]	[0.40, 0.48]
PEH/CS	0.28 ±0.01	0.29 ±0.00	0.28 ±0.01	0.27 ±0.01
	[0.26, 0.30]	[0.29, 0.30]	[0.25, 0.32]	[0.25, 0.31]
NOH/CS	0.13 ±0.01	0.15 ±0.01	0.13 ±0.01	0.12 ±0.01
	[0.11, 0.15]	[0.14, 0.17]	[0.11, 0.16]	[0.10, 0.15]
PPH/CS	0.27 ±0.01	0.26 ±0.01	0.27 ±0.01	0.26 ±0.01
	[0.25, 0.30]	[0.24, 0.27]	[0.24, 0.31]	[0.24, 0.28]
PPL/CS	0.30 ±0.02	0.26 ±0.02	0.27 ±0.02	0.27 ±0.02
	[0.25, 0.34]	[0.24, 0.29]	[0.23, 0.30]	[0.23, 0.31]

### Synopsis of Malagasy members of the *Nesomyrmex madecassus* species-group

*flavus* Csősz & Fisher, sp. n.

*gibber* (Donisthorpe, 1946)

*madecassus* (Forel, 1892)

*nitidus* Csősz & Fisher, sp. n.

### Key to workers of the *Nesomyrmex madecassus* group species

**Table:** 

1	Mesothoracic hump conspicuous. Mauritius only	***gibber***
–	Mesothoracic hump absent. Madagascar	**2**
2	Dark brown to black	***madecassus*** (dark phenotype)
–	Yellow to light brown	**3**
3	Postocular area (PoOC) longer relative to cephalic width including compound eyes (CW): CW/PoOC >1.85 (min. 1.77, max. 2.07), [5-95% percentiles: min. 1.85, max. 2.01]	***madecassus*** (ocher phenotype)
–	Postocular area (PoOC) shorter relative to cephalic width including compound eyes (CW): CW/PoOC < 1.85 (min. 1.52, max. 1.89), [5-95% percentiles: min. 1.60, max. 1.84]	**4**
4	Occur at higher altitudes/elevations: mean = 1190 m, [min. 200, max. 1755 m]. For precise morphological separations a discriminant D2 (+0.0847*SL -0.0625*MW -15.038) function is available. D2 scores (n = 61) = +3.09 [+0.98, +5.33]	***flavus***
–	Distributed in lower elevations: mean = 383 m, [min. 10, max. 1550 m]. For precise morphological separations a discriminant D2 function is available. D2 scores (n = 79) = -2.39 [-4.63, +0.19]	***nitidus***

#### Nesomyrmex flavus

Taxon classificationAnimaliaHymenopteraFormicidae

Csősz & Fisher sp. n.

http://zoobank.org/FD4F716F-93CB-42AB-95F9-26C76A65386B

Figs 4–6 , Table 2

##### Type material investigated.

**Holotype: CASENT0393113**, collection code: BLF36563: MADAGASCAR, Prov. Toliara, Anosy Region, Anosyenne Mts, 31.2 km NW Manantenina, N -24.13894, E 47.06804, alt 1125 m, B.L. Fisher, F.A. Esteves et al., 2_26_2015, (1w, CAS);

**Paratypes**: Five workers, four gynes and two males with the same label data with the holotype under CASENT codes: **CASENT0393110**, collection code: BLF36563 (1w, 1q, CAS); **CASENT0393111**, collection code: BLF36563 (1w, 1q, CAS); **CASENT0393112**, collection code: BLF36563 (1w, 1q, CAS); **CASENT0393113**, collection code: BLF36563 (1q, CAS); **CASENT0393114**, collection code: BLF36563 (1w, 1m, CAS); **CASENT0393115**, collection code: BLF36563 (1w, 1m, CAS)

##### Material examined.

**MADAGASCAR: CASENT0119576** (collection code: BLF14982, 1w, CAS, CASENT0119576): Prov. Fianarantsoa, Parc National Befotaka-Midongy, Papango 28.5 km S Midongy-Sud, Mount Papango, N -23.84083, E 46.9575, alt 1250 m, B.L. Fisher et al., 11_17_2006; **CASENT0121813** (collection code: BLF15514, 1w, CAS, CASENT0121813): Prov. Toliara, Forêt Ivohibe 55.0 km N Tolagnaro, N -24.569, E 47.204, alt 200 m, B.L. Fisher et al., 12_3_2006; **CASENT0129841** (collection code: BLF15450, 1w, CAS): Prov. Toliara, Forêt Ivohibe 55.0 km N Tolagnaro, N -24.569, E 47.204, alt 200 m, B.L. Fisher et al., 12_2_2006; **CASENT0127625** (collection code: BLF01972, 2w, CAS): Prov. Antsiranana, Prov.Antsiranana R.S. Manongarivo 17.3 km 218° SW Antanambao, N -14.02167, E 48.4183, alt 1580 m, B.L. Fisher, 10_27_1998; **CASENT0127641** (collection code: BLF00748, 1w, CAS): Prov. Fianarantsoa, 43 km S Ambalavao, Rés. Andringitra, N -22.23333, E 47, alt 800 m, B.L. Fisher, 10_6_1993; **CASENT0127642** (collection code: BLF01023, 1w, CAS): Prov. Toamasina, 6.9 km NE Ambanizana, Ambohitsitondroina, N -13.56667, E 50, alt 1080 m, B.L. Fisher, 12_9_1993; **CASENT0127643** (collection code: BLF00740, 1w, CAS): Prov. Fianarantsoa, 45 km S Ambalavao, N -22.21667, E 47.0167, alt 720 m, B.L. Fisher, 10_1_1993; **CASENT0127646** (collection code: BLF01751, 2w, CAS): Prov. Fianarantsoa, R.S. Ivohibe, 6.5 km ESE Ivohibe, N -22.49667, E 46.955, alt 1575 m, B.L. Fisher (Sylvain), 10_24_1997; **CASENT0141281** (collection code: BLF20452, 1w, CAS), **CASENT0141284** (collection code: BLF20457, 2w, CAS): Prov. Fianarantsoa, Parc naturel communautaire, 26.8 km SW Ambositra, N -20.775, E 47.1836, alt 1755 m, B.L. Fisher et al., 5_20_2008; **CASENT0148667** (collection code: BLF21477, 1w, CAS), **CASENT0149902** (collection code: BLF21545, 1w, CAS): Prov. Toliara, Réserve Spéciale Kalambatritra, Betanana, N -23.4144, E 46.459, alt 1360 m, B.L. Fisher et al., 2_8_2009; **CASENT0148948** (collection code: BLF21630, 1w, CAS), **CASENT0148980** (collection code: BLF21600, 1w, CAS), **CASENT0150536** (collection code: BLF21632, 1w, CAS), Prov. Toliara, Réserve Spéciale Kalambatritra, Ampanihy, N -23.4635, E 46.4631, alt 1270 m, B.L. Fisher et al., 2_9_2009; **CASENT0151026** (collection code: BLF21705, 1w, CAS): Prov. Toliara, Réserve Spéciale Kalambatritra, Ampanihy, N -23.463, E 46.4706, alt 1269 m, B.L. Fisher et al., 2_10_2009; **CASENT0189276** (collection code: BLF01626, 3w, CAS, CASENT0189276): Prov. Fianarantsoa, 29 km SSW Ambositra, Ankazomivady, N -20.77667, E 47.165, alt 1700 m, B.L. Fisher, 1_14_1998; **CASENT0227040** (collection code: BLF1972(8), 1w, CAS), **CASENT0227042** (collection code: BLF01014, 1w, CAS), **CASENT0227044** (collection code: BLF01014, 1w, CAS): Prov. Toamasina, 6.9 km NE Ambanizana, Ambohitsitondroina, N -13.56667, E 50, alt 1080 m, B.L. Fisher, 12_9_1993; **CASENT0409543** (collection code: BLF02398, 1w, CAS), **CASENT0409544** (collection code: BLF02398, 1w, CAS), **CASENT0409547** (collection code: BLF02398, 1w, CAS), **CASENT0409582** (collection code: BLF02451, 2w, CAS), **CASENT0409583** (collection code: BLF02421, 2w, CAS), **CASENT0409585** (collection code: BLF02451, 2w, CAS), **CASENT0409586** (collection code: BLF02435, 2w, CAS), **CASENT0409587** (collection code: BLF02435, 1w, CAS), **CASENT0409588** (collection code: BLF02465, 2w, CAS), **CASENT0409590** (collection code: BLF02465, 2w, CAS), **CASENT0409591** (collection code: BLF02447, 1w, CAS): Prov. Antananarivo, 3 km 41° NE Andranomay, 11.5 km 147° SSE Anjozorobe, N -18.47333, E 47.96, alt 1300 m, Fisher, Griswold et al., 12_5_2000; **CASENT0418989** (collection code: BLF03695, 1w, CAS), **CASENT0418990** (collection code: BLF03695, 1w, CAS), **CASENT0418996** (collection code: BLF03695, 2w, CAS): Prov. Antananarivo, Réserve Spéciale d’Ambohitantely, Forêt d Ambohitantely, 20.9 km 72° NE d Ankazobe, N -18.22528, E 47.2868, alt 1410 m, Fisher, Griswold et al., 4_17_2001; **CASENT0447182** (collection code: BLF05014, 1w, CAS), **CASENT0447195** (collection code: BLF05014, 1w, CAS), **CASENT0447265** (collection code: BLF05014, 1w, CAS), **CASENT0447266** (collection code: BLF05014, 1w, CAS), **CASENT0447267** (collection code: BLF05014, 1w, CAS), **CASENT0447269** (collection code: BLF05014, 1w, CAS): Prov. Toliara, Parc National d’Andohahela, Col du Sedro, 3.8 km 113° ESE Mahamavo, 37.6 km 341° NNW Tolagnaro, N -24.76389, E 46.7517, alt 900 m, Fisher-Griswold Arthropod Team, 1_21_2002; **CASENT0477187** (collection code: BLF02543, 2w, CAS),; **CASENT0488066** (collection code: BLF02544, 1w, CAS): Prov. Antananarivo, 3 km 41° NE Andranomay, 11.5 km 147° SSE Anjozorobe, N -18.47333, E 47.96, alt 1300 m, Griswold et al., 12_5_2000; **CASENT0494106** (collection code: BLF09806, 1w, CAS),; **CASENT0494153** (collection code: BLF09859, 2w, CAS): Prov. Antsiranana, Forêt de Binara, 9.4 km 235° SW Daraina, N -13.26333, E 49.6, alt 1100 m, B.L. Fisher, 12_5_2003;

##### Description of workers.

Body color: yellow. Body color pattern: Body concolorous. Absolute cephalic size: 602 [533, 699]. Cephalic length vs. Maximum width of head capsule (CL/CWb): 1.26 [1.19, 1.36]. Postocular distance vs. cephalic length (PoOc/CL): 0.48 [0.46, 0.50]. Postocular sides of cranium contour frontal view orientation: converging posteriorly. Postocular sides of cranium contour frontal view shape: convex. Vertex contour line in frontal view shape: straight; slightly concave. Vertex sculpture: main sculpture inconspicuous, ground sculpture smooth. Gena contour line in frontal view shape: convex. Genae contour from anterior view orientation: converging; strongly converging. Gena sculpture: NOT CODED. Concentric carinae laterally surrounding antennal foramen: present. Eye length vs. absolute cephalic size (EL/CS): 0.25 [0.23, 0.27]. Frontal carina distance vs. absolute cephalic size (FRS/CS): 0.30 [0.28, 0.32]. Longitudinal carinae on median region of frons: present. Longitudinal carinae on medial region of frons shape: variable. Smooth median region on frons: present. Antennomere count: 12. Scape length vs. absolute cephalic size (SL/CS): 0.80 [0.76, 0.83]. Median clypeal notch: variable. Ground sculpture of submedian area of clypeus: present. Median carina of clypeus: absent. Lateral carinae of clypeus: present. Metanotal depression: variable. Dorsal region of mesosoma sculpture: fine areolate ground sculpture, superimposed by dispersed rugae. Lateral region of pronotum sculpture: areolate ground sculpture, main sculpture dispersed costate. Mesopleuron sculpture: fine areolate ground sculpture, superimposed by dispersed rugulae. Metapleuron sculpture: fine areolate ground sculpture, superimposed by dispersed rugulae. Petiole width vs. absolute cephalic size (PEW/CS): 0.22 [0.21, 0.24]. Anterior profile of petiolar node contour line in lateral view shape: concave. Dorso-caudal petiolar profile contour line in lateral view shape: convex; strongly convex. Dorsal region of petiole sculpture: ground sculpture smooth, main sculpture absent. Postpetiole width vs. absolute cephalic size (PPW/CS): 0.35 [0.33, 0.40]. Dorsal region of postpetiole sculpture: ground sculpture smooth, main sculpture dispersed rugose; ground sculpture smooth, main sculpture absent.

**Figures 4–6.**
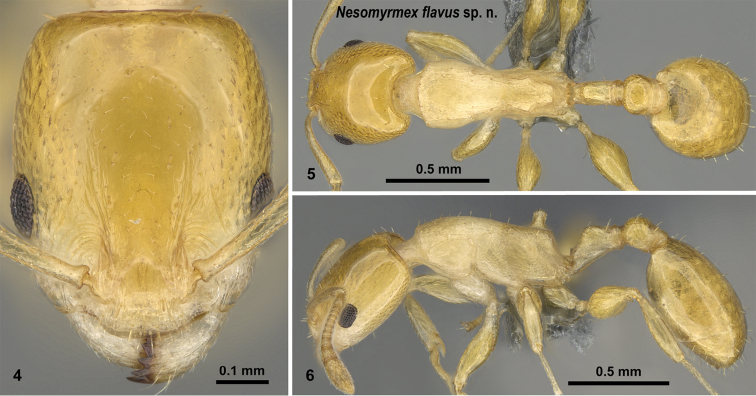
*Nesomyrmex flavus* sp. n. holotype worker (CASENT0393113). Head in full-face view (**4**), dorsal view of the body (**5**), lateral view of the body (**6**).

##### Diagnosis.

Workers of *Nesomyrmex flavus* cannot be confused with *Nesomyrmex gibber* because the conspicuous mesothoracic hump which is a diagnostic character of the latter species is absent in *Nesomyrmex flavus* workers. This species can be easily separated from dark phenotypes of *Nesomyrmex madecassus* by color: the dark madecassus phenotypes are dark brown but the workers of *Nesomyrmex flavus* are light yellow. Morphometric ratio (PoOC/CW) and discriminant D4 function helps to separate *Nesomyrmex flavus* from ocher *madecassus* phenotypes; further details are given in diagnosis under *Nesomyrmex madecassus*.

The workers of this species are the most similar to that of *Nesomyrmex nitidus*. The elevational distribution of the two species may provide hints useful for separation (Fig. 3) but the ranges broadly overlap. These taxa represent true cryptic species which cannot be identified based on qualitative characters (i.e. sculpture, shape or color), and their overlapping range means ratios cannot be used for identification. Therefore, only a discriminant D2 function with a greatly reduced character set (D2 = +0.0847*SL -0.0625*MW -15.038) yields complete separation (morphometric data are in micrometer):

*flavus* D2 (n = 61) = +3.09 [+0.98, +5.33]

*nitidus* D2 (n = 79) = -2.39 [-4.63, +0.19]

For now, this remains the simplest method available to separate workers of these two taxa, but in the future, when more information about these species has been accumulated, we hope to find a reliable and easy-to-use diagnostic trait.

##### Biology and distribution.

This species is known to occur in Madagascar’s rain forests at high altitudes between 200 and 1755 m, mean: 1190 m (Fig. 3). This species is known to forage in low vegetation and nests can often be found in dead twigs. This species has occasionly been collectied in leaf litter (leaf mold, rotten wood), or in rotten tree stumps.

#### Nesomyrmex gibber

Taxon classificationAnimaliaHymenopteraFormicidae

(Donisthorpe, 1946)

Figs 7–9 , Table 2

##### Type material investigated.

**Holotype**: “Ireneopone gibber, H. Donistorphe, 1946 TYPE” Mauritius, Calebasses Mt., 22. X. 1944, No 72 leg. R. Mamet (1w, BMNH, London, U.K., CASENT0102303), [type specimen was morphometrically not investigated, AntWeb images were used for comparison]

##### Material examined.

**MAURITIUS: MCZENT0578588** (1w, MCZ), **MCZENT0578589** (1w, MCZ), **MCZENT0578590** (1w, MCZ), **MCZENT0578591** (1w, MCZ), **MCZENT0578592** (1w, MCZ), **MCZENT0578593** (1w, MCZ, CASENT0178539): Le Pouce Mt., N -20.19, E 57.52, alt 700–800 m, W.L. Brown, 1_29_1977; **CASENT0922013**, (collection code: PSW10502, 1w, PSW, CASENT0922013): Basin Blanc, Mauritius, N -20.45, E 57.4667, alt 500 m, P.S. Ward, 5_06_1989.

##### Description of workers.

Body color: brown. Body color pattern: Body concolorous. Absolute cephalic size: 724 [655, 752]. Cephalic length vs. Maximum width of head capsule (CL/CWb): 1.17 [1.14, 1.18]. Postocular distance vs. cephalic length (PoOc/CL): 0.41 [0.39, 0.42]. Postocular sides of cranium contour frontal view orientation: converging posteriorly. Postocular sides of cranium contour frontal view shape: convex. Vertex contour line in frontal view shape: straight; slightly concave. Vertex sculpture: main sculpture absent, ground sculpture areolate. Gena contour line in frontal view shape: convex. Genae contour from anterior view orientation: converging; strongly converging. Gena sculpture: ground sculpture areolate, main sculpture absent. Concentric carinae laterally surrounding antennal foramen: absent. Eye length vs. absolute cephalic size (EL/CS): 0.25 [0.24, 0.26]. Frontal carina distance vs. absolute cephalic size (FRS/CS): 0.33 [0.32, 0.34]. Longitudinal carinae on median region of frons: absent. Smooth median region on frons: present. Antennomere count: 12. Scape length vs. absolute cephalic size (SL/CS): 0.80 [0.78, 0.82]. Median clypeal notch: present. Ground sculpture of submedian area of clypeus: present. Median carina of clypeus: absent. Lateral carinae of clypeus: absent. Metanotal depression: present. Dorsal region of mesosoma sculpture: ground sculpture areolate, main sculpture absent. Lateral region of pronotum sculpture: ground sculpture areolate, main sculpture absent. Mesopleuron sculpture: ground sculpture areolate, main sculpture absent. Metapleuron sculpture: ground sculpture areolate, main sculpture absent. Petiole width vs. absolute cephalic size (PEW/CS): 0.21 [0.20, 0.23]. Anterior profile of petiolar node contour line in lateral view shape: concave. Dorso-caudal petiolar profile contour line in lateral view shape: strongly convex. Dorsal region of petiole sculpture: ground sculpture areolate, main sculpture absent. Postpetiole width vs. absolute cephalic size (PPW/CS): 0.30 [0.27, 0.32]. Dorsal region of postpetiole sculpture: ground sculpture areolate, main sculpture absent.

**Figures 7–9.**
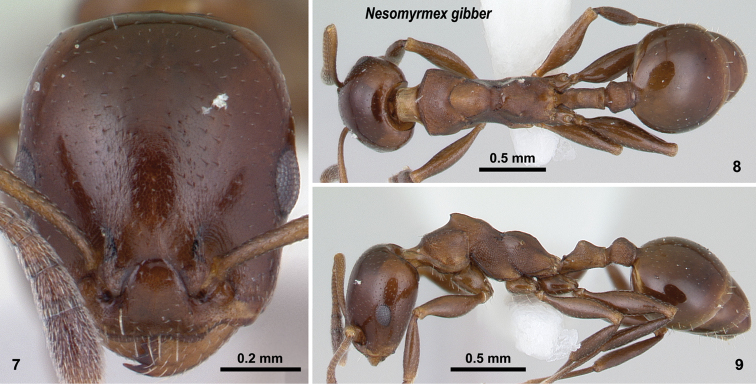
*Nesomyrmex gibber* non-type worker (CASENT0178540). Head in full-face view (**7**), dorsal view of the body (**8**), lateral view of the body (**9**).

##### Diagnosis.

This species is easily distinguished from all the other taxa treated in this revisionary work by the presence of the conspicuous mesothoracic hump on the mesosoma of workers (Fig. 9).

##### Biology and distribution.

Endemic to Mauritius island. Occur in rainforests in higher altitude between 500 and 800 meters, (mean = 714 m). This species van be collected on low vegetation and in dead stems.

#### Nesomyrmex madecassus

Taxon classificationAnimaliaHymenopteraFormicidae

(Forel, 1892)

**Figures 10–12.**
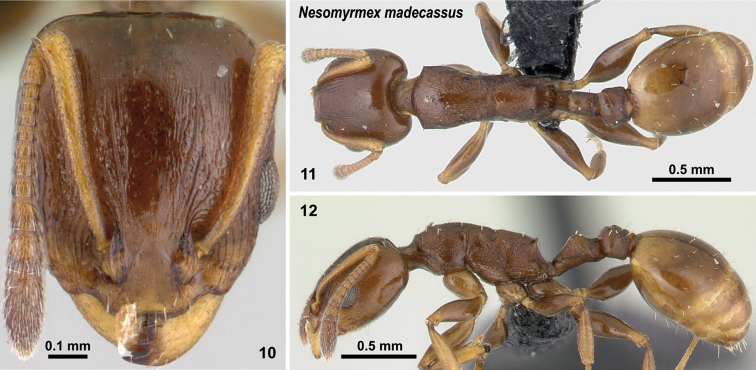
*Nesomyrmex madecassus* non-type worker (CASENT0487142). Head in full-face view (**10**), dorsal view of the body (**11**), lateral view of the body (**12**).

Figs 10–12 , Table 2

##### Type material investigated.

**Syntype workers**: CASENT0101667, (collection code: ANTC3246), CASENT0101687 (collection code: ANTC3247): [MADAGASCAR, Prov.] Antsiranana, Forêt d’ Andrangoloaka [Antananarivo, -18.91 N, 47.55 E], Madagascar (Sikora)”, (CASENT0101667, CASENT0101687, MHNG);

##### Material examined.

**Dark (wild type) phenotype: MADAGASCAR: BLF1626** (collection code: BLF1626, 3w, CAS), **BLF1626(16)** (collection code: BLF1626(16), 2w, CAS): Fianarantsoa, 29 km SSW Ambositra, Ankazomivady, N -20.77667, E 47.165, alt 1700 m, B.L. Fisher, 1_14_1998; **CASENT0040463** (collection code: BLF09324, 1w, CAS): Antsiranana, Parc National de Marojejy, 25.4 km 30° NNE Andapa, 10.9 km 311° NW Manantenina, N -14.445, E 49.735, alt 2000 m, B.L. Fisher, 11_23_2003; **CASENT0048441** (collection code: BLF10513, 2w, CAS): Toamasina, Analamay, N -18.80623, E 48.33707, alt 1068 m, Malagasy ant team, 3_21_2004; **CASENT0049029** (collection code: BLF10704, 2w, CAS): Toamasina, Torotorofotsy, N -18.87082, E 48.34737, alt 1070 m, Malagasy ant team, 3_29_2004; **CASENT0051178** (collection code: BLF10689, 2w, CAS): Toamasina, Torotorofotsy, N -18.87082, E 48.34737, alt 1070 m, Malagasy ant team, 3_28_2004; **CASENT0058844** (collection code: BLF11968, 1w, CAS): Toamasina, Forêt Ambatovy, 14.3 km 57° Moramanga, N -18.85083, E 48.32, alt 1075 m, B.L. Fisher, 4_12_2005; **CASENT0071372** (collection code: BLF13809, 1w, CAS), **CASENT0071382** (collection code: BLF13794, 1w, CAS), **CASENT0071385** (collection code: BLF13811, 1w, CAS), **CASENT0071427** (collection code: BLF13798, 1w, CAS), **CASENT0071431** (collection code: BLF13800, 1w, CAS), **CASENT0071437** (collection code: BLF13785, 1w, CAS), **CASENT0071439** (collection code: BLF13775, 1w, CAS): Fianarantsoa, Parc National d’Andringitra, Plateau d’Andohariana, 39.8 km 204° Ambalavao, N -22.18767, E 46.90083, alt 2150 m, B.L. Fisher et al., 4_16_2006; **CASENT0097142** (collection code: MA-01-01D-01, 1w, CAS), **CASENT0097143** (collection code: MA-01-01D-01, 1w, CAS): Diego-Suarez, Parc National Montagne d’Ambre [Petit Lac road], N -12.52028, E 49.17917, alt 1125 m, Irwin, Schlinger, Harin’H, 1_25_2001; **CASENT0111762** (collection code: MG-29-06, 1w, CAS): Fianarantsoa, Miandritsara Forest, 40Km S of Ambositra, N -20.79267, E 47.17567, alt 822 m, Rin’ha, Mike, 1_5_2005; **CASENT0112777** (collection code: MA-02-09C-60, 1w, CAS): Fianarantsoa, Belle Vue trail, Ranomafana National Park, Fianarantsoa Prov., N -21.2665, E 47.42017, alt 1020 m, R. Harin’Hala, 5_4_2003; **CASENT0114296** (collection code: MA-02-09D-06, 1w, CAS): Fianarantsoa, JIRAMA water works near river, Ranomafana National Park, Fianarantsoa Prov., N -21.2485, E 47.45217, alt 690 m, R. Harin’Hala, 12_6_2001; **CASENT0118350** (collection code: MG-29-04, 1w, CAS): Fianarantsoa, Miandritsara Forest, 40Km S of Ambositra, N -20.79267, E 47.17567, alt 822 m, Rin’ha, Mike, 11_14_2004; **CASENT0122389** (collection code: BLF17425, 1w, CAS), **CASENT0125701** (collection code: BLF17430, 1w, CAS), **CASENT0125880** (collection code: BLF17420, 1w, CAS): Antananarivo, Kaloy, N -18.58998, E 47.65102, alt 1423 m, B.L. Fisher et al., 4_27_2007; **CASENT0125872** (collection code: BLF17314, 1w, CAS): Antananarivo, Ambohimanga, N -18.76125, E 47.56447, alt 1361 m, B.L. Fisher et al., 4_26_2007; **CASENT0127628** (collection code: BLF01989, 1w, CAS): Antsiranana, R.S. Manongarivo, 20.4 km 219° SW Antanambao, N -14.04667, E 48.40167, alt 1860 m, B.L. Fisher, 11_3_1998; **CASENT0127627** (collection code: BLF01972, 3w, CAS): Antsiranana, Prov. Antsiranana R.S. Manongarivo 17.3 km 218° SW Antanambao, N -14.02167, E 48.41833, alt 1580 m, B.L. Fisher, 10_27_1998; **CASENT0127629** (collection code: MA-01-01A-01, 1w, CAS): Diego-Suarez, Parc National Montagne d’Ambre [1st campsite], N -12.51444, E 49.18139, alt 960 m, Irwin, Schlinger, Harin’H, 1_21_2001; **CASENT0127630** (collection code: ANTC8395, 2w, CAS): Antsiranana, RNI Marojejy, 11 km NW Manantenina, N -14.45, E 49.73333, alt 1875 m, E.L. Quinter, 11_13_1996; **CASENT0127636** (collection code: ASS(03)-1, 2w, CAS): Fianarantsoa, Rés. Andringitra, Plateau d’Andohariana, base of Pic d’Ivangomena, N -22.2, E 46.9, alt 2050 m, Goodman leg., 9_3_1995; **CASENT0127647** (collection code: BLF01755, 2w, CAS): Fianarantsoa, 8.0 km NE Ivohibe, N -22.42167, E 46.89833, alt 1200 m, B.L. Fisher (Sylvain), 11_3_1997; **CASENT0136019** (collection code: BLF18083, 1w, CAS): Antsiranana, Parc National Montagne d’Ambre, Lac maudit, N -12.58502, E 49.15147, alt 1250 m, B.L. Fisher et al., 11_13_2007; **CASENT0142139** (collection code: BLF20488, 1w, CAS), **CASENT0142157** (collection code: BLF20480, 1w, CAS): Fianarantsoa, Parc naturel communautaire, 28.5 km SW Ambositra, N -20.78414, E 47.16699, alt 1780 m, B.L. Fisher et al., 5_21_2008; **CASENT0212236** (collection code: BLF26175, 1w, CAS): Antsiranana, Parc National Montagne d’Ambre, N -12.51778, E 49.17957, alt 1000 m, B.L. Fisher et al., 3_6_2011; **CASENT0227041** (collection code: BLF01989, 1w, CAS, CASENT0227040), **CASENT0227050** (collection code: BLF01989, 1w, CAS), **CASENT0227052** (collection code: BLF01989, 1w, CAS): Antsiranana, R.S. Manongarivo, 20.4 km 219° SW Antanambao, N -14.04667, E 48.40167, alt 1860 m, B.L. Fisher, 11_3_1998; **CASENT0230550** (collection code: BLF26169, 1w, CAS), **CASENT0245051** (collection code: BLF26169, 1w, CAS, CASENT0409551): Antsiranana, Parc National Montagne d’Ambre, N -12.51778, E 49.17957, alt 1000 m, B.L. Fisher et al., 3_6_2011; **CASENT0274650** (collection code: BLF28278, 1w, CAS): Antananarivo, Réserve Speciale d’Ambohitantely, N -18.22444, E 47.2774, alt 1490 m, B.L. Fisher et al., 3_9_2012; **CASENT0275845** (collection code: BLF28339, 1w, CAS): Antananarivo, Mandraka Park, N -18.9019, E 47.90786, alt 1360 m, B.L. Fisher et al., 3_11_2012; **CASENT0409550** (collection code: BLF02398, 1w, CAS), **CASENT0409551** (collection code: BLF02398, 1w, CAS): Antananarivo, 3 km 41° NE Andranomay, 11.5 km 147° SSE Anjozorobe, N -18.47333, E 47.96, alt 1300 m, Fisher, Griswold et al., 12_5_2000; **CASENT0486880** (collection code: BLF09120, 1w, CAS), **CASENT0487129** (collection code: BLF09369, 2w, CAS), **CASENT0487141** (collection code: BLF09412, 2w, CAS), **CASENT0487143** (collection code: BLF09412, 2w, CAS), **CASENT0487177** (collection code: BLF09372, 2w, CAS), **CASENT0487210** (collection code: BLF09416, 2w, CAS), **CASENT0487357** (collection code: BLF09315, 4w, CAS): Antsiranana, Parc National de Marojejy, 25.7 km 32° NNE Andapa, 10.3 km 314° NW Manantenina, N -14.445, E 49.74167, alt 1575 m, B.L. Fisher, 11_22_2003; MCZENT0576254 (1w, MCZ): Antsiranana, 10k NE Antananarivo lac Alarobie, G.D. Alpert, 3_10_1991;

**Ocher phenotype: CASENT0119572** (collection code: BLF15013, 1w, CAS), **CASENT0119575** (collection code: BLF15088, 1w, CAS), **CASENT0119580** (collection code: BLF15089, 1w, CAS): Fianarantsoa, Parc National Befotaka-Midongy, Papango 28.5km S Midongy-Sud, Mount Papango, N -23.84083, E 46.9575, alt 1250 m, B.L. Fisher et al., 11_19_2006; **CASENT0149638** (collection code: BLF21513, 1w, CAS, CASENT0149638): Toliara, Réserve Spéciale Kalambatritra, N -23.4185, E 46.4583, alt 1365 m, B.L. Fisher et al., 2_8_2009; **CASENT0148666** (collection code: BLF21476, 1w, CAS): Toliara, Réserve Spéciale Kalambatritra, Betanana, N -23.4144, E 46.459, alt 1360 m, B.L. Fisher et al., 2_8_2009; **CASENT0192603** (collection code: BLF01626, 1w, CAS): Fianarantsoa, 29 km SSW Ambositra, Ankazomivady, N -20.77667, E 47.165, alt 1700 m, B.L. Fisher, 1_14_1998; **CASENT0141296** (collection code: BLF20465, 1w, CAS): Fianarantsoa, Parc naturel communautaire, 28.5 km SW Ambositra, N -20.78414, E 47.16699, alt 1780 m, B.L. Fisher et al., 5_21_2008; **CASENT0127621** (collection code: BLF01626, 4w, CAS): Fianarantsoa, 29 km SSW Ambositra, Ankazomivady, N -20.77667, E 47.165, alt 1700 m, B.L. Fisher, 1_14_1998; **CASENT0127626** (collection code: BLF01989, 1w, CAS): Antsiranana, R.S. Manongarivo, 20.4 km 219° SW Antanambao, N -14.04667, E 48.40167, alt 1860 m, B.L. Fisher, 11_3_1998;

##### Description of workers.

Body color: brown; black; rarely yellow. Body color pattern: Body concolorous. If yellow, body concolorous, clava, femora and 1st gastral tergite darker. Absolute cephalic size: 692 [616, 763]. Cephalic length vs. Maximum width of head capsule (CL/CWb): 1.18 [1.13, 1.22]. Postocular distance vs. cephalic length (PoOc/CL): 0.46 [0.43, 0.48]. Postocular sides of cranium contour frontal view orientation: converging posteriorly. Postocular sides of cranium contour frontal view shape: convex. Vertex contour line in frontal view shape: straight; slightly concave. Vertex sculpture: main sculpture inconspuous, ground sculpture smooth. Gena contour line in frontal view shape: convex. Genae contour from anterior view orientation: converging. Gena sculpture: rugoso-reticulate with feeble areolate ground sculpture. Concentric carinae laterally surrounding antennal foramen: present. Eye length vs. absolute cephalic size (EL/CS): 0.26 [0.24, 0.28]. Frontal carina distance vs. absolute cephalic size (FRS/CS): 0.31 [0.29, 0.33]. Longitudinal carinae on median region of frons: absent. Smooth median region on frons: present. Antennomere count: 12. Scape length vs. absolute cephalic size (SL/CS): 0.78 [0.72, 0.82]. Median clypeal notch: variable. Ground sculpture of submedian area of clypeus: present. Median carina of clypeus: absent. Lateral carinae of clypeus: present. Metanotal depression: variable. Dorsal region of mesosoma sculpture: areolate ground sculpture, superimposed by dispersed rugae. Lateral region of pronotum sculpture: areolate ground sculpture, main sculpture dispersed costate. Mesopleuron sculpture: ground sculpture areolate, main sculpture absent. Metapleuron sculpture: ground sculpture areolate, main sculpture absent. Petiole width vs. absolute cephalic size (PEW/CS): 0.22 [0.19, 0.24]. Anterior profile of petiolar node contour line in lateral view shape: concave. Dorso-caudal petiolar profile contour line in lateral view shape: convex. Dorsal region of petiole sculpture: ground sculpture areolate, main sculpture absent. Postpetiole width vs. absolute cephalic size (PPW/CS): 0.35 [0.29, 0.39]. Dorsal region of postpetiole sculpture: ground sculpture areolate, main sculpture absent.

##### Diagnosis.

Workers of this species differ from that of *Nesomyrmex gibber* by having no mesothoracic hump, and from *Nesomyrmex flavus* sp. n. and *Nesomyrmex nitidus* sp. n. by its dark brown color versus the light yellow hue of the two latter species.

The dark color in *madecassus* populations is dominant across the entire known distributional area, and comprises ~95% of the examined material. However, a rare, lighter-colored *madecassus* phenotype (ocher phenotype) was also found in a few localities. There is no evidence, other than color, that would suport heterospecifity of these two discrete phenotypes of *Nesomyrmex madecassus* workers and no correlation was found between elevational cline and color. Only one mixed sample is known to include both ocher and dark phenotype. Ocher *madecassus* phenotypes are darker than the majority of *Nesomyrmex flavus* and *Nesomyrmex nitidus* workers and also differ from the latter species by having brown femora and a dark patch on the first gastral tergite.

*Nesomyrmex madecassus* workers (including ocher phenotypes) can be separated from those of *Nesomyrmex flavus* and *Nesomyrmex nitidus* using the ratio of postocular area to cephalic width including compound eyes (PoOC/CW), which yielded only three misclassified cases:

*madecassus* (n = 84) = 1.92 (1.77, 2.07), [5-95% percentiles: min. 1.85, max. 2.01]

*flavus* (n = 61) = 1.73 (1.53, 1.89), [5-95% percentiles: min. 1.60, max. 1.84]

*nitidus* (n = 79) = 1.73 (1.52, 1.85), [5-95% percentiles: min. 1.63, max. 1.83]

A more precise means to separate ocher *madecassus* phenotype from workers of *Nesomyrmex flavus* and *Nesomyrmex nitidus* may be necessary. In these cases, a discriminant D4 funtion (D4 = +0.0511*PoOC -0.0486*CW -0.0702*PEW +0.0435*PEL +8.3829) provides a moderately time consuming classification tool yielding non-overlapping ranges between *madecassus* workers and that of *flavus* and *nitidus* (morphometric data are given in micrometers):

*madecassus* D4 (n = 84) = -1.70 [-4.61, 0.26]

*flavus* D4 (n = 61) = +2.39 [0.42, 5.02]

*nitidus* D4 (n = 79) = +3.18 [0.51, 5.98]

##### Biology and distribution.

This species is known to occur in Madagascar’s rain forests at very high altitudes between 690 and 2150 m, mean: 1538 m (Fig. 3). This species is known to forage in low vegetation, nests can often be found in dead twigs, or rarely in leaf litter (leaf mold, rotten wood), or in rotten tree stumps.

#### Nesomyrmex nitidus

Taxon classificationAnimaliaHymenopteraFormicidae

Csősz & Fisher sp. n.

http://zoobank.org/F0E325CD-99C1-4CB0-817D-4D7EC060AD8F

**Figures 13–15.**
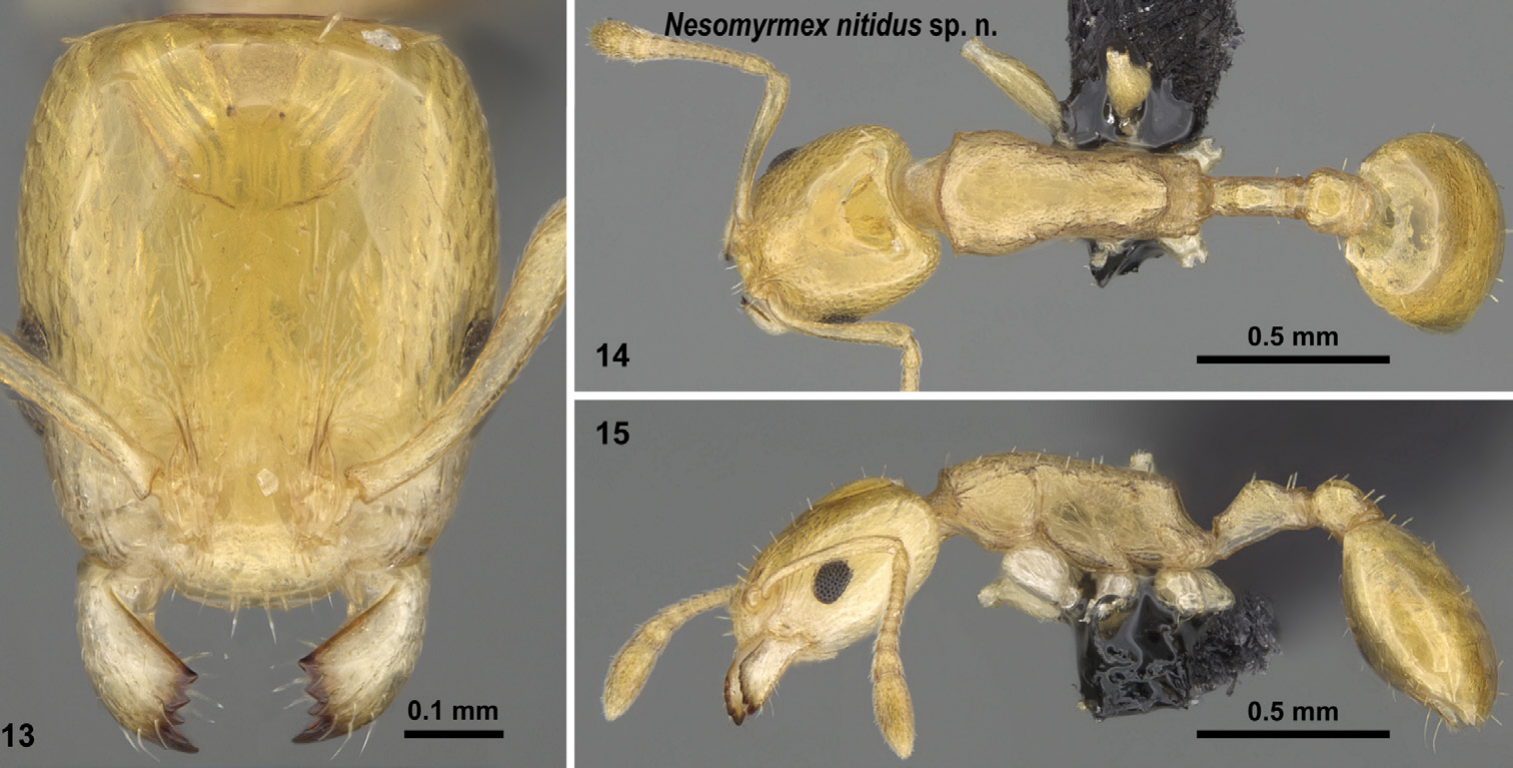
*Nesomyrmex nitidus* sp. n. holotype worker (CASENT0163151). Head in full-face view (**13**), dorsal view of the body (**14**), lateral view of the body (**15**).

Figs 13–15 , Table 2

##### Type material investigated.

**Holotype: CASENT0163151**, collection code: BLF24792: MADAGASCAR, Prov. Toamasina, Réserve Spéciale Ambatovaky, Sandrangato river, N -16.81753, E 49.29498, alt 360 m, B.L. Fisher et al. 2_25_2010, (1w, CAS);

**Paratypes**: two workers and one gyne from the same locality under CASENT codes: **CASENT0163112**, collection code: BLF24794: MADAGASCAR, Prov. Toamasina, Réserve Spéciale Ambatovaky, Sandrangato river, N -16.81753, E 49.29498, alt 360 m, B.L. Fisher et al.2_25_2010, (1w, CAS); **CASENT0162145**, collection code: BLF24570: MADAGASCAR, Prov. Toamasina, Réserve Spéciale Ambatovaky, Sandrangato river, N -16.7633, E 49.26692, alt 520 m, B.L.Fisher et al.2_26_2010, (1w, CAS); **CASENT0161445**, collection code: BLF25001: MADAGASCAR, Prov. Toamasina, Réserve Spéciale Ambatovaky, Sandrangato river, N -16.81209, E 49.29216, alt 460 m, B.L. Fisher et al.2_22_2010, (1q, CAS);

##### Material examined.

**MADAGASCAR: BLF1888(24)** (collection code: BLF01888, 1w, CAS): Prov. Antsiranana, R.S. Manongarivo, 12.8 km 228° SW Antanambao, N -13.97667, E 48.42333, alt 780 m, B.L. Fisher, 10_12_1998; **CASENT0059931** (collection code: BLF12392, 1w, CAS): Prov. Fianarantsoa, 7.6 km 122º Kianjavato, Forêt Classée Vatovavy, N -21.4, E 47.94, alt 175 m, B.L. Fisher et al., 6_6_2005; **CASENT0067500** (collection code: BLF12687, 1w, CAS): Prov. Toamasina, Parc National Mananara-Nord, 7.1 km 261° Antanambe, N -16.455, E 49.7875, alt 225 m, B.L. Fisher et al., 11_16_2005; **CASENT0068002** (collection code: BLF12780, 2w, CAS): Prov. Toamasina, Res. Ambodiriana, 4.8 km 306° Manompana, along Manompana river, N -16.67233, E 49.70117, alt 125 m, B.L.Fisher et al., 11_18_2005; **CASENT0076214** (collection code: BLF09620, 1w, CAS): Prov. Antsiranana, Forêt de Binara, 7.5 km 230° SW Daraina, N -13.255, E 49.61667, alt 375 m, B.L. Fisher, 12_2_2003; **CASENT0077523** (collection code: BLF09713, 1w, CAS): Prov. Antsiranana, Forêt de Binara, 9.1km 233° SW Daraina, N -13.26333, E 49.60333, alt 650-800 m, B.L. Fisher, 12_4_2003; **CASENT0107046** (collection code: BLF11562, 1w, CAS), **CASENT0107052** (collection code: BLF11562, 1w, CAS): Prov. Antsiranana, Forêt Ambato, 26.6 km 33° Ambanja, N -13.4645, E 48.55167, alt 150 m, B.L. Fisher, 12_9_2004; **CASENT0107060** (collection code: BLF11610, 1w, CAS): Prov. Antsiranana, Forêt Ambato, 26.6 km 33° Ambanja, N -13.4645, E 48.55167, alt 150 m, B.L. Fisher, 12_10_2004; **CASENT0110675** (collection code: BLF11220, 1w, CAS): Prov. Antsiranana, Ambondrobe, 41.1km 175° Vohemar, N -13.71533, E 50.10167, alt 10 m, B.L. Fisher, 11_30_2004; **CASENT0129913** (collection code: BLF15100, 1w, CAS): Prov. Toliara, Parc National Andohahela, Col de Tanatana, 33.3km NW Tolagnaro, N -24.7585, E 46.85367, alt 275 m, B.L. Fisher et al., 11_22_2006; **CASENT0136588** (collection code: BLF18628, 1w, CAS): Prov. Antsiranana, Forêt d’Ampombofofo, N -12.09949, E 49.33874, alt 25 m, B.L. Fisher et al., 11_21_2007; **CASENT0151045** (collection code: BLF22399, 1w, CAS): Prov. Toamasina, Parc National de Zahamena, Sahavorondrano River, N -17.75257, E 48.85725, alt 765 m, B.L. Fisher et al., 2_23_2009; **CASENT0151511** (collection code: BLF23080, 1w, CAS): Prov. Mahajanga, Réserve forestière Beanka, 50.2 km E Maintirano, N -17.88756, E 44.47265, alt 153 m, B.L.Fisher et al., 10_31_2009; **CASENT0151914** (collection code: BLF22603, 1w, CAS, CASENT0151914): Prov. Antsiranana, Betaolana Forest, along Bekona River, N -14.52996, E 49.44039, alt 880 m, B.L. Fisher et al., 3_5_2009; **CASENT0152470** (collection code: BLF22141, 1w, CAS, CASENT0152470): Prov. Toamasina, Parc National de Zahamena, Tetezambatana forest, near junction of Nosivola and Manakambahiny Rivers, N -17.74298, E 48.72936, alt 860 m, B.L. Fisher et al., 2_19_2009; **CASENT0155948** (collection code: BLF22797, 1w, CAS): Prov. Mahajanga, Réserve forestière Beanka, 50.2 km E Maintirano, N -18.02649, E 44.05051, alt 250 m, B.L. Fisher et al., 10_19_2009; **CASENT0156676** (collection code: BLF22969, 1w, CAS): Prov. Mahajanga, Réserve forestière Beanka, 53.6 km E Maintirano, N -18.04014, E 44.53394, alt 272 m, B.L. Fisher et al., 10_25_2009; **CASENT0162145** (collection code: BLF24570, 2w, CAS): Prov. Toamasina, Réserve Spéciale Ambatovaky, Sandrangato river, N -16.7633, E 49.26692, alt 520 m, B.L. Fisher et al., 2_22_2010; **CASENT0162819** (collection code: BLF24484, 1w, CAS): Prov. Toamasina, Réserve Spéciale Ambatovaky, Sandrangato river, N -16.76912, E 49.26704, alt 475 m, B.L. Fisher et al., 2_21_2010; **CASENT0163112** (collection code: BLF24794, 1w, CAS), **CASENT0163151** (collection code: BLF24792, 1w, CAS): Prov. Toamasina, Réserve Spéciale Ambatovaky, Sandrangato river, N -16.81753, E 49.29498, alt 360 m, B.L. Fisher et al., 2_25_2010; **CASENT0205731** (collection code: BLF25790, 1w, CAS): Prov. Toliara, Makay Mts., N -21.25864, E 45.16412, alt 500 m, B.L. Fisher et al., 12_8_2010; **CASENT0208609** (collection code: BLF25261, 1w, CAS): Prov. Toliara, Makay Mts., N -21.21985, E 45.32396, alt 500 m, B.L. Fisher et al., 11_25_2010; **CASENT0245134** (collection code: BLF26356, 1w, CAS): Prov. Antananarivo, Ankalalahana, N -19.00659, E 47.1122, alt 1375 m, B.L. Fisher et al., 3_29_2011; **CASENT0261123** (collection code: BLF27634, 2w, CAS): Prov. Fianarantsoa, Andrambovato along river Tatamaly, N -21.51082, E 47.40992, alt 1063 m, B.L. Fisher et al., 10_24_2011; **CASENT0419627** (collection code: BLF04344, 1w, CAS): Prov. Mahajanga, Parc National Tsingy de Bemaraha, 2.5 km 62° ENE Bekopaka, Ankidrodroa River, N -19.13222, E 44.81467, alt 100 m, Fisher-Griswold Arthropod Team, 11_11_2001; **CASENT0419848** (collection code: BLF04434, 1w, CAS), **CASENT0419849** (collection code: BLF04434, 1w, CAS): Prov. Mahajanga, Parc National Tsingy de Bemaraha, 10.6 km ESE 123° Antsalova, N -18.70944, E 44.71817, alt 150 m, Fisher-Griswold Arthropod Team, 11_16_2001; **CASENT0422571** (collection code: BLF03132, 1w, CAS): Prov. Antsiranana, Montagne des Français, 7.2 km 142° SE Antsiranana (=Diego Suarez), N -12.32278, E 49.33817, alt 180 m, Fisher, Griswold et al., 2_22_2001; **CASENT0422585** (collection code: BLF03426, 1w, CAS), **CASENT0422593** (collection code: BLF03426, 1w, CAS), **CASENT0422597** (collection code: BLF03426, 2w, CAS): Prov. Antsiranana, Nosy Be, Réserve Naturelle Intégrale de Lokobe, 6.3 km 112° ESE Hellville, N -13.41933, E 48.33117, alt 30 m, Fisher, Griswold et al., 3_19_2001; **CASENT0422629** (collection code: BLF02859, 2w, CAS), **CASENT0422651** (collection code: BLF02859, 1w, CAS): Prov. Antsiranana, Réserve Spéciale de l’Ankarana, 22.9 km 224° SW Anivorano Nord, N -12.90889, E 49.10983, alt 80 m, Fisher, Griswold et al., 2_10_2001; **CASENT0422673** (collection code: BLF02660, 1w, CAS): Prov. Antsiranana, Réserve Spéciale d’Ambre, 3.5 km 235° SW Sakaramy, N -12.46889, E 49.24217, alt 325 m, Fisher, Griswold et al., 1_26_2001; **CASENT0422690** (collection code: BLF03426, 1w, CAS), **CASENT0422691** (collection code: BLF03426, 2w, CAS): Prov. Antsiranana, Nosy Be, Réserve Naturelle Intégrale de Lokobe, 6.3 km 112° ESE Hellville, N -13.41933, E 48.33117, alt 30 m, Fisher, Griswold et al., 3_19_2001; **CASENT0443282** (collection code: BLF04234, 2w, CAS), **CASENT0443283** (collection code: BLF04234, 2w, CAS), **CASENT0443284** (collection code: BLF04234, 2w, CAS): Prov. Mahajanga, Parc National Tsingy de Bemaraha, 3.4 km 93° E Bekopaka, Tombeau Vazimba, N -19.14194, E 44.828, alt 50 m, Fisher-Griswold Arthropod Team, 11_6_2001; **CASENT0474737** (collection code: BLF06448, 1w, CAS): Prov. Mahajanga, Parc National de Namoroka, 9.8 km 300° WNW Vilanandro, N -16.46667, E 45.35, alt 140 m, Fisher, Griswold et al., 11_4_2002; **CASENT0484804** (collection code: BLF07511, 2w, CAS), **CASENT0484870** (collection code: BLF07511, 2w, CAS), **CASENT0484900** (collection code: BLF07511, 1w, CAS): Prov. Toliara, Parc National de Zombitse, 19.8 km 84° E Sakaraha, N -22.84333, E 44.71, alt 770 m, Fisher, Griswold et al., 2_5_2003; **CASENT0490345** (collection code: BLF07384, 1w, CAS), **CASENT0490346** (collection code: BLF07384, 2w, CAS): Prov. Fianarantsoa, Forêt d’Analalava, 29.6 km 280° W Ranohira, N -22.59167, E 45.12833, alt 700 m, Fisher, Griswold et al., 2_1_2003; **CASENT0490719** (collection code: BLF07703, 2w, CAS), **CASENT0491357** (collection code: BLF07762, 2w, CAS), **CASENT0491554** (collection code: BLF07293, 1w, CAS): Prov. Fianarantsoa, Forêt d’Atsirakambiaty, 7.6 km 285° WNW Itremo, N -20.59333, E 46.56333, alt 1550 m, Fisher, Griswold et al., 1_22_2003; **CASENT0491364** (collection code: BLF07761, 2w, CAS), **CASENT0492591** (collection code: BLF07652, 2w, CAS), **CASENT0492611** (collection code: BLF07652, 2w, CAS), **CASENT0492612** (collection code: BLF07652, 2w, CAS): Prov. Fianarantsoa, Parc National d’Isalo, Sahanafa River, 29.2 km 351° N Ranohira, N -22.31333, E 45.29167, alt 500 m, Fisher, Griswold et al., 2_10_2003; **CASENT0494326** (collection code: BLF09951, 1w, CAS): Prov. Antsiranana, Forêt de Bekaraoka, 6.8 km 60° ENE Daraina, N -13.16667, E 49.71, alt 150 m, B.L. Fisher, 12_8_2003; **CASENT0495109** (collection code: BLF08147, 2w, CAS): Prov. Toamasina, Montagne d’Anjanaharibe, 18.0 km 21° NNE Ambinanitelo, N -15.18833, E 49.615, alt 470 m, Fisher, Griswold et al., 3_8_2003; **CASENT0498718** (collection code: BLF10016, 2w, CAS), **CASENT0498721** (collection code: BLF10016, 2w, CAS): Prov. Antsiranana, Forêt d’Ampondrabe, 26.3 km 10° NNE Daraina, N -12.97, E 49.7, alt 175 m, B.L. Fisher, 12_10_2003.

##### Description of workers.

Body color: yellow. Body color pattern: Body concolorous. Absolute cephalic size: 496 [460, 574]. Cephalic length vs. maximum width of head capsule (CL/CWb): 1.23 [1.16, 1.35]. Postocular distance vs. cephalic length (PoOc/CL): 0.48 [0.46, 0.50]. Postocular sides of cranium contour frontal view orientation: converging posteriorly. Postocular sides of cranium contour frontal view shape: convex. Vertex contour line in frontal view shape: straight; slightly concave. Vertex sculpture: main sculpture inconspuous, ground sculpture smooth. Gena contour line in frontal view shape: convex. Genae contour from anterior view orientation: converging. Gena sculpture: rugoso-reticulate with feeble areolate ground sculpture. Concentric carinae laterally surrounding antennal foramen: present. Eye length vs. absolute cephalic size (EL/CS): 0.26 [0.23, 0.27]. Frontal carina distance vs. absolute cephalic size (FRS/CS): 0.31 [0.29, 0.33]. Longitudinal carinae on median region of frons: absent. Smooth median region on frons: present. Antennomere count: 12. Scape length vs. absolute cephalic size (SL/CS): 0.74 [0.69, 0.78]. Median clypeal notch: variable. Ground sculpture of submedian area of clypeus: smooth; present. Median carina of clypeus: variable. Lateral carinae of clypeus count: present. Metanotal depression: variable. Dorsal region of mesosoma sculpture: areolate ground sculpture, superimposed by dispersed rugae. Lateral region of pronotum sculpture: ground sculpture areolate, main sculpture absent. Mesopleuron sculpture: ground sculpture areolate, main sculpture absent. Metapleuron sculpture: ground sculpture areolate, main sculpture absent. Petiole width vs. absolute cephalic size (PEW/CS): 0.22 [0.19, 0.24]. Anterior profile of petiolar node contour line in lateral view shape: concave. Dorso-caudal petiolar profile contour line in lateral view shape: convex. Dorsal region of petiole sculpture: ground sculpture smooth, main sculpture absent. Postpetiole width vs. absolute cephalic size (PPW/CS): 0.33 [0.30, 0.36]. Dorsal region of postpetiole sculpture: ground sculpture smooth, main sculpture dispersed rugose.

##### Diagnosis.

Workers of *Nesomyrmex nitidus* cannot be confused with *Nesomyrmex gibber* because the conspicuous mesothoracic hump that is a diagnostic character of the latter species is absent in *Nesomyrmex nitidus* workers. This species also can be easily separated from dark phenotypes of *Nesomyrmex madecassus* based on color: the dark madecassus phenotypes are dark brown but the workers of *Nesomyrmex nitidus* are light yellow. Morphometric ratio (PoOC/CW) and discriminant D4 function helps to separate *Nesomyrmex nitidus* from ocher *madecassus* phenotypes; further details are given in Diagnosis under *Nesomyrmex madecassus*.

The workers of this species are the most similar to that of *Nesomyrmex flavus*. The broadly overlapping elevational distribution as well as qualitative and quantitative traits of *Nesomyrmex flavus* and *Nesomyrmex nitidus* workers hamper easy separation. A simplified discriminant D2 function with a greatly reduced character set for safe separation is provided in the diagnosis section of *Nesomyrmex flavus*.

##### Biology and distribution.

This species typically occurs in Madagascar’s rain forests at lower altitudes between 10 and 1550 meter, mean: 383 m (Fig. 3). This species is known to forage in low vegetation, nests can often be found in dead twigs, stems above ground or rarely in rotten logs at higher elevations.

## Supplementary Material

XML Treatment for Nesomyrmex flavus

XML Treatment for Nesomyrmex gibber

XML Treatment for Nesomyrmex madecassus

XML Treatment for Nesomyrmex nitidus
